# Type 2 diabetes mellitus complicated by acquired reactive perforating collagenosis: A case report and literature review

**DOI:** 10.1097/MD.0000000000050728

**Published:** 2026-09-11

**Authors:** Li Li, Hanpeng Wu, Liuyan Zhao

**Affiliations:** a Department of Endocrinology and Metabolism, Hangzhou Third People’s Hospital, Hangzhou, Zhejiang Province, China.

**Keywords:** acquired reactive perforating collagenosis, histopathology, type 2 diabetes mellitus, Van Gieson staining

## Abstract

**Rationale:**

Acquired reactive perforating collagenosis (ARPC) is a perforating skin disorder characterized by transepidermal elimination of degenerated collagen. It is often associated with systemic diseases, such as diabetes and chronic kidney disease. Due to its diverse clinical manifestations, ARPC is often misdiagnosed or overlooked in clinical practice.

**Patient concerns:**

A 66-year-old female with a 22-year history of type 2 diabetes (T2DM) was admitted to the hospital due to poor blood glucose control. She had been experiencing itching and a rash for nearly 3 months and had previously been treated at another hospital, but the response was unsatisfactory. Physical examination revealed erythema, papules, and nodules on the extensor surfaces of both lower extremities.

**Diagnoses:**

The patient had a confirmed diagnosis of T2DM. Histopathological examination of the skin lesion revealed collagen fibers penetrating through the epidermis, which were positive for Van Gieson staining. Therefore, the final diagnosis was T2DM complicated by ARPC.

**Interventions:**

Oral antihistamines and topical corticosteroid cream were given along with blood glucose control.

**Outcomes:**

After the aforementioned treatment, the patient’s blood glucose level was well controlled, the skin lesions gradually healed, and the pruritus significantly subsided.

**Lessons:**

This case suggests that the occurrence of ARPC may be closely related to persistent hyperglycemia. ARPC skin lesions can be regarded as one of the cutaneous manifestations of underlying metabolic diseases. When such lesions appear or worsen, clinicians should comprehensively evaluate the patient’s overall condition, particularly blood glucose control. Treatment requires a dual-pronged approach, including active correction of systemic triggers such as hyperglycemia, as well as local intervention for skin lesions.

## 1. Introduction

Reactive perforating collagenosis (RPC) is a very rare, chronic perforating dermatosis first described by Mehregan et al in 1967.^[1]^ It is characterized by transepidermal elimination of altered collagen fibers in the superficial dermis after minor trauma, such as scratching, rubbing, or insect bites, leading to the formation of characteristic keratotic papules.^[2]^ Based on age of onset and etiology, this disease is clinically classified into hereditary and acquired types.^[2]^ The hereditary type is typically seen in childhood and may have an autosomal recessive inheritance. By contrast, acquired reactive perforating collagenosis (ARPC) typically affects adults and is closely linked to underlying systemic diseases, particularly diabetes mellitus and chronic renal disease.^[3]^ ARPC is very rare and often misinterpreted or overlooked because of its clinical resemblance to other entities such as prurigo nodularis and keratotic eczema. This article is aimed at reporting a case of type 2 diabetes mellitus (T2DM) complicated by ARPC and at conducting a literature review to raise clinicians’ awareness of this condition.

### 1.1. Case presentation

The patient is a 66-year-old woman with a 22-year history of T2DM. Over the past 3 months, she has experienced polyuria, dry mouth, and polydipsia without any apparent cause, and her self-monitored blood glucose levels have fluctuated between 5 and 22 mmol/L. She was admitted to the hospital on December 8, 2025. In addition, the patient has a 12-year history of medical conditions, including coronary heart disease (CHD), atrial fibrillation, and hypertension, and underwent radiofrequency ablation for atrial fibrillation in 2013. Three months prior to admission, she had erythematous macules and papules in both lower extremities with intractable pruritus with no identifiable triggers. After being examined at a local hospital, she was diagnosed with pruritus and was treated with various antihistamines and topical corticosteroids. However, the response to treatment was poor, and the skin lesions progressively enlarged, accompanied by erosion and ulceration. She denied any history of food or drug allergies. Personal, marital, reproductive, and family histories were unremarkable.

### 1.2. Physical examination

**Figure 1.**
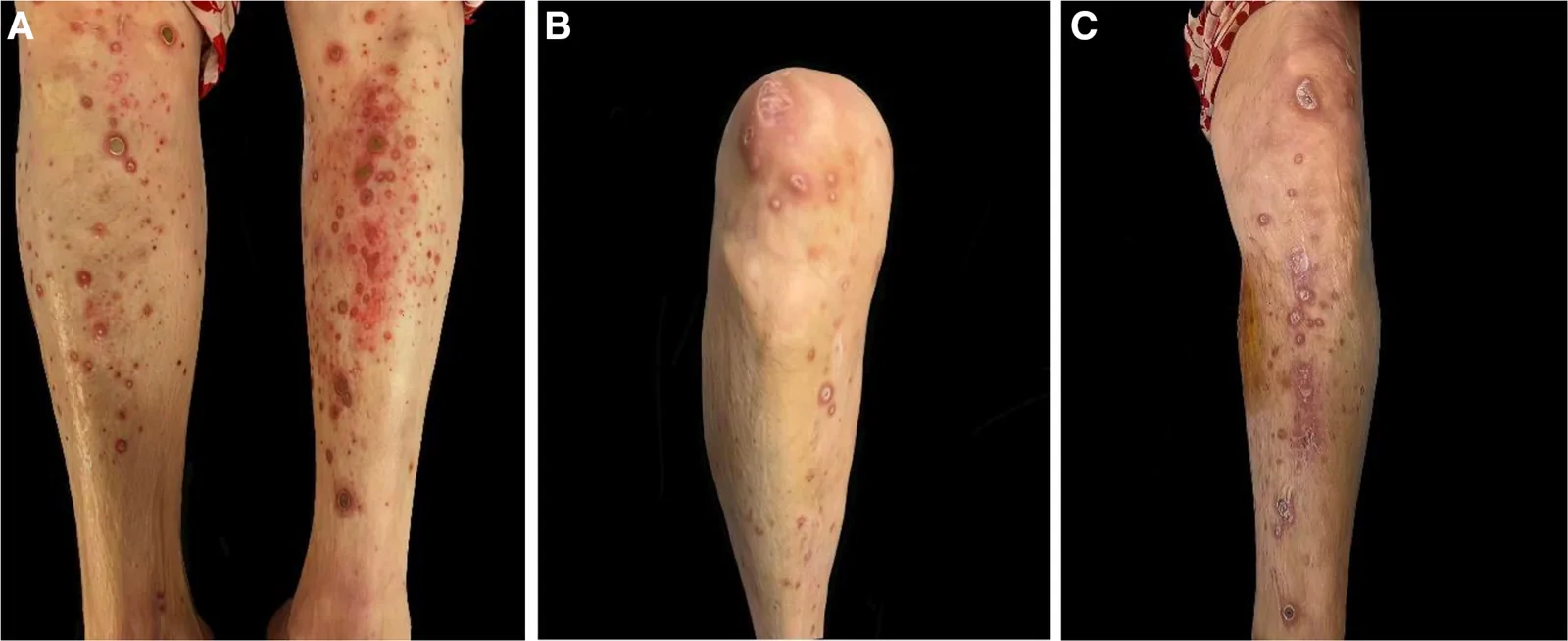
Comparison of the patient’s skin condition before and after treatment. (A) Before treatment: scattered erythema, papules, and nodules with central depression and yellowish-brown crusts were present on the extensor surfaces of both lower limbs, with some lesions coalescing into patches. (B) and (C) After treatment: erythema had faded, the rash had partially resolved, the lesion area was reduced, and eroded surfaces were largely crusted over and healed.

The heart rhythm was irregular with variable intensity of the first heart sound; no pathological murmurs were audible. Examination of the lungs and abdomen revealed no significant abnormalities. Skin Examination: Multiple erythematous patches, papules, and nodules – ranging in size from mung bean to broad bean – were scattered over the extensor surfaces of both lower limbs (Fig. 1A). The lesions had slightly raised borders with central umbilication and were covered by yellowish-brown crusts, with some coalescing into plaques.

### 1.3. Investigations

Glycemic assessment showed glycated hemoglobin A1c (HbA1c) 9.9%, fructosamine 2.48 mmol/L, fasting blood glucose 11.84 mmol/L, and 2-hour postprandial blood glucose 9.68 mmol/L. A lipid profile revealed total cholesterol 5.35 mmol/L, low-density lipoprotein 3.58 mmol/L, high-density lipoprotein 1.44 mmol/L, and triglycerides 1.18 mmol/L. Bacterial culture of secretions was negative. Complete blood count, urinalysis, liver and renal function tests, cardiac enzyme profile, B-type natriuretic peptide, urine albumin-to-creatinine ratio, human immunodeficiency virus antibody screening, thyroid function tests, and tumor markers were all within normal limits.

Carotid artery ultrasound showed intima-media thickening of the right common carotid artery and atherosclerotic plaques at the bifurcations of both common carotid arteries and of the brachiocephalic trunk. Lower extremity arterial ultrasound revealed atherosclerotic plaques in the arteries of both lower limbs. Brain magnetic resonance angiography and diffusion-weighted imaging were suggestive of cerebral arteriosclerosis. Chest computed tomography demonstrated multiple solid and ground-glass nodular opacities in both lungs. Abdominal and urinary tract ultrasounds were unremarkable. Electrocardiography (ECG) revealed atrial fibrillation.

## 2. Diagnosis

The patient’s diagnosis of T2DM was well established. During this hospitalization, the diagnosis and treatment of skin lesions were another priority, for which we requested a dermatological consultation. According to expert opinion, we should focus on the differential diagnosis of ARPC and neutrophilic dermatosis, and rule out infectious dermatitis. A skin biopsy for histopathological examination and direct immunofluorescence (DIF) was recommended to establish a definitive diagnosis.

**Figure 2.**
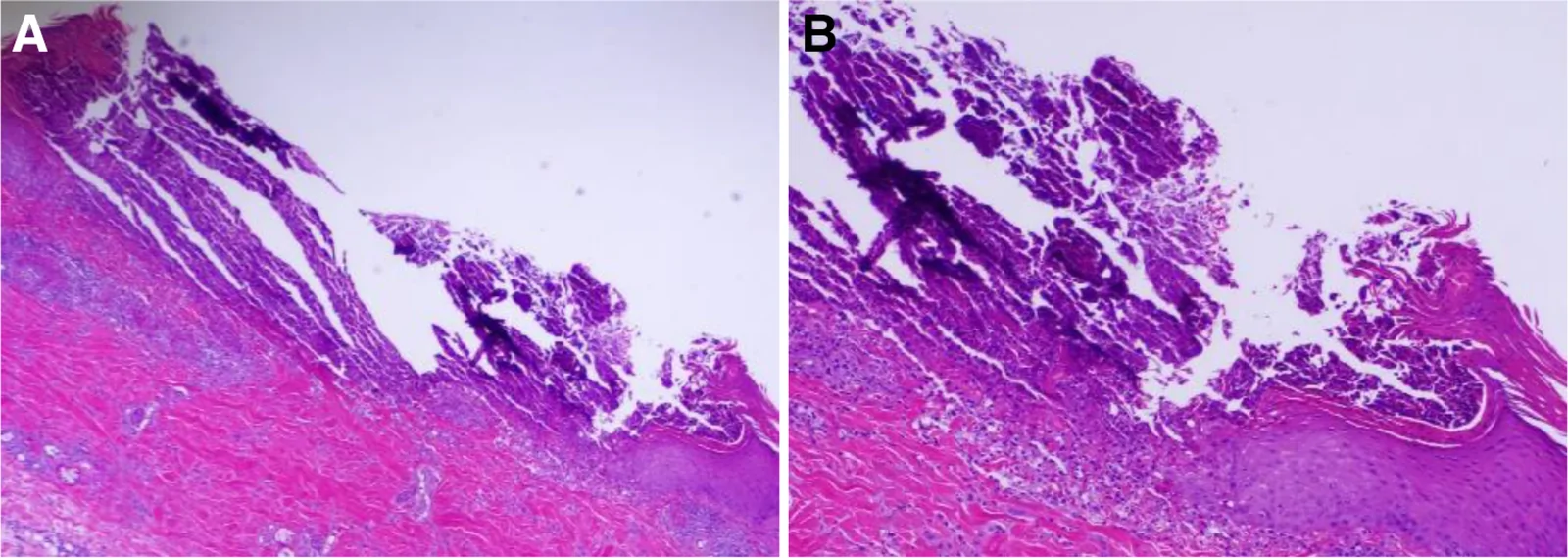
Histopathology of the left lower limb skin lesion demonstrates epidermal ulceration with a dense inflammatory cell infiltrate, suspected extrusion of collagen fibers toward the epidermis, mild acanthosis of the epidermis flanking the ulcer, and a sparse perivascular infiltrate of lymphocytes and histiocytes in the superficial dermis. (A) HE × 40, (B) HE × 100.

**Figure 3.**
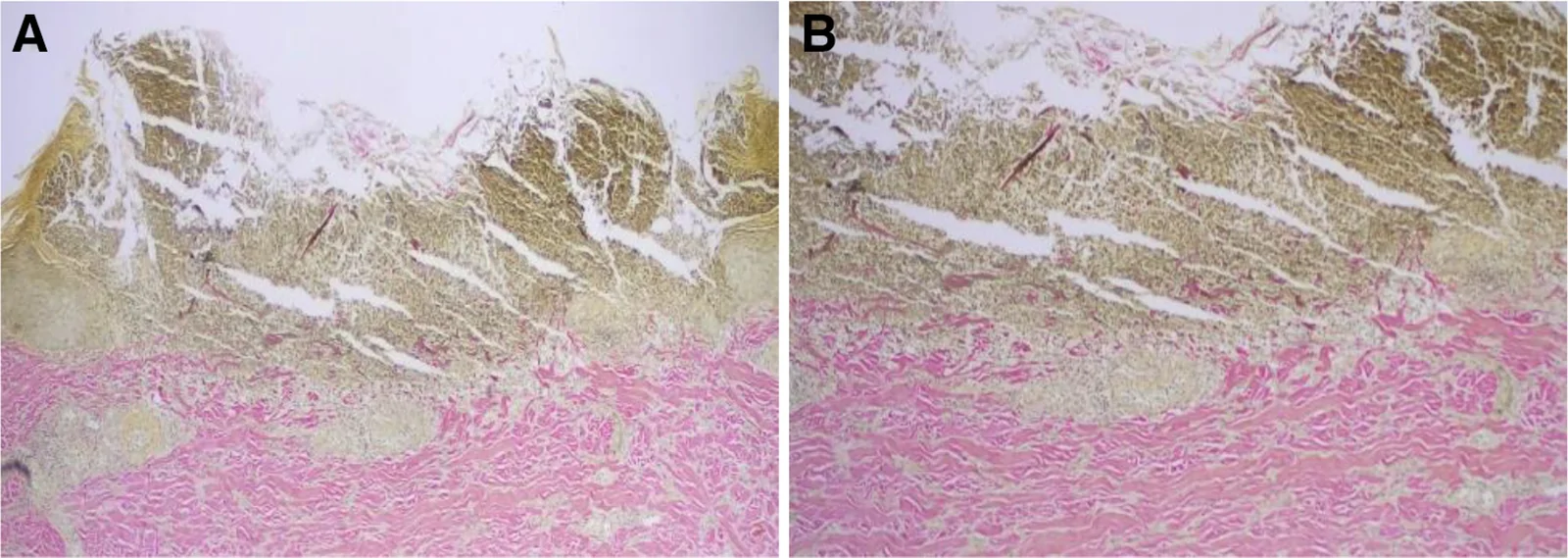
Histopathology of the left lower limb skin lesion: Van Gieson staining demonstrates collagen fibers penetrating the epidermis (A) Van Gieson × 40, (B) Van Gieson × 100.

**Figure 4.**
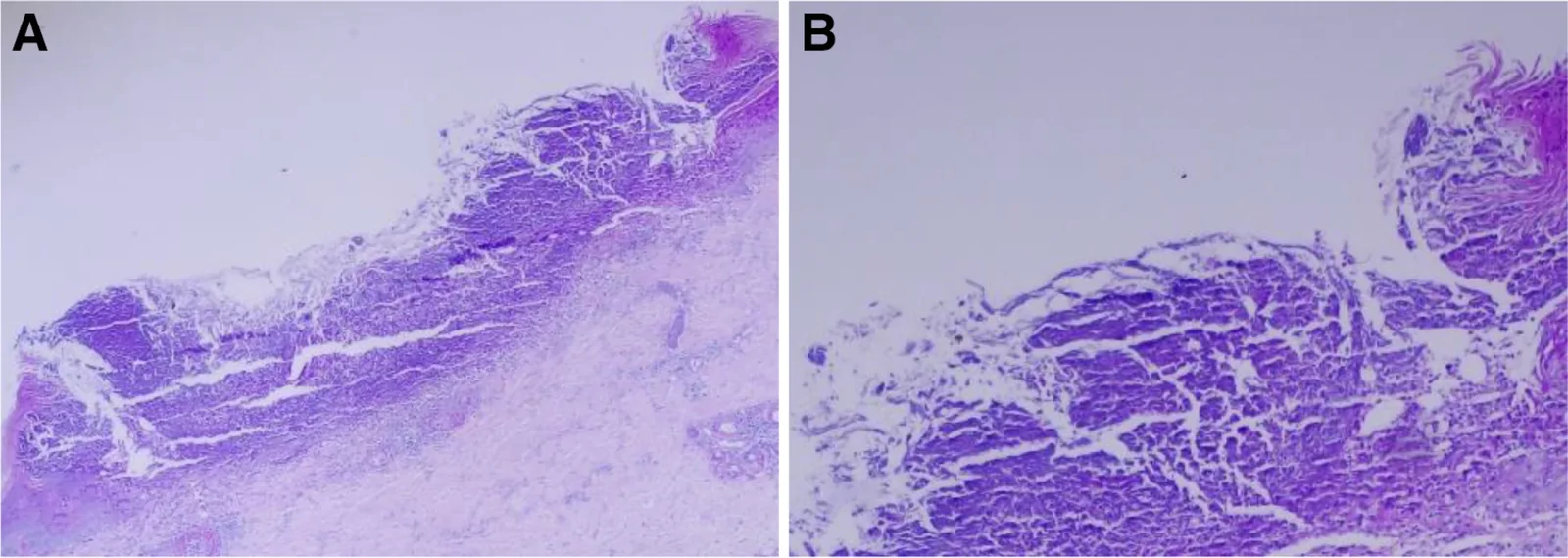
Histopathology of the left lower limb skin lesion: Periodic acid–Schiff (PAS) staining is negative (A) PAS × 40, (B) PAS × 100.

Subsequently, a biopsy was taken from a skin lesion on the left lower extremity and sent for histopathology, special staining, and DIF. The pathology results showed ulceration with inflammatory infiltration in the epidermis, and transepidermal elimination of collagen fibers was suspected. Mild epidermal thickening was visible on both sides of the ulcer. In the superficial dermis, mild perivascular infiltration of lymphocytes and histiocytes was observed (Fig. 2). Van Gieson (VG) stain was positive for collagen fibers penetrating the epidermis (Fig. 3), and periodic acid-Schiff (PAS) stain was negative (Fig. 4). DIF was negative for IgG, IgM, IgA, and C3, which essentially rules out immune complex-mediated skin diseases.

The final diagnosis was T2DM complicated by ARPC.

### 2.1. Treatment

A stepwise approach to glycemic management was used. There was significant glucose lability (5–22 mmol/L), for which we started continuous subcutaneous insulin infusion (CSII) with insulin aspart for rapid stabilization. Upon improvement, the regimen changed to twice-daily insulin aspart 30/70, acarbose, and metformin.

As for the skin lesions, a symptomatic approach was adopted, including antiallergic, anti-inflammatory, and infection-preventive therapies, before arriving at a definite diagnosis. Systemic antihistamine therapy was given using chlorphenamine maleate injection (10mg, once at night, intramuscularly) and ebastine capsules (10mg, once daily). For local treatment, wet compresses containing dexamethasone injection (15mg) in boric acid–chloramphenicol solution (500mL) were applied, followed by alternating application of compound clotrimazole cream and erythromycin ointment for anti-inflammatory and antiseptic action and to reduce pruritus. Following a definitive histopathological diagnosis, the treatment was simplified to systemic antihistamines (chlorphenamine maleate and ebastine) and topical halometasone-triclosan cream.

### 2.2. Outcome and follow-up

After comprehensive treatment, both blood glucose levels and skin lesions improved. Blood glucose levels were well controlled, with fasting glucose of 7.0–8.6 mmol/L and 2-hour postprandial glucose of 7.9–10.9 mmol/L. A marked improvement in skin condition was noted, including darkening of erythema, partial regression of the rash, reduction in size of skin lesions, and essentially complete crusting and healing of previously eroded areas (Fig. 1B and 1C). At the 1-month telephone follow-up, the majority of the lesions had healed, and self-monitored fasting and 2-hour postprandial glucose were in the range of 6–8 mmol/L and 8–10 mmol/L, respectively. A summary of the diagnosis and treatment is provided in Table 1.

**Table 1:** Summary of diagnosis and treatment for T2DM complicated by ARPC.

Category	Content
Key clinical findings	Poor glycemic control; characteristic skin lesions on both lower limbs (papules, nodules, umbilical depression, yellowish-brown crusts)
Investigations	Hyperglycemia (FBG 11.84 mmol/L, 2h-PBG 9.68 mmol/L); dyslipidemia (LDL 3.58 mmol/L); bacterial culture of secretions (−); atherosclerosis (carotid, lower extremity arteries, and cerebral); atrial fibrillation (ECG)
Histopathology	Transepidermal elimination of collagen fibers; VG (+), PAS (−), DIF (−)
Differential diagnoses	Neutrophilic dermatosis, infectious dermatitis
Therapeutic interventions	Glycemic control: CSII intensive therapy → insulin aspart 30 bid + acarbose + metformin; Skin lesions: empirical antihistamines + topical anti-inflammatory and infection prevention →simplified to systemic antihistamines + topical halometasone/triclosan cream after diagnosis
Clinical outcomes	Glycemic control stabilized; skin lesions gradually crusted and healed completely at 1-month follow-up

2h-PBG = 2-hour postprandial blood glucose, bid = twice daily, CSII = continuous subcutaneous insulin infusion, DIF = direct immunofluorescence, ECG = electrocardiogram, FBG = fasting blood glucose, LDL = low-density lipoprotein, PAS = periodic acid-Schiff stain, VG = Van Gieson stain.

## 3. Discussion

### 3.1. Pathogenesis

The peak age of onset for ARPC is around 50–60 years, and the incidence in males and females is similar.^[4,5]^ The exact pathogenesis of ARPC is still not fully understood, but it is believed to result from multiple factors. It is frequently associated with systemic conditions, such as diabetes mellitus, chronic renal failure, hypertension, malignancies, thyroid dysfunction, acquired immunodeficiency syndrome, and hepatic disorders.^[6,7]^ Studies have shown that ARPC is frequently associated with diabetes (especially in patients with poor glycemic control),^[8,9]^ and hyperglycemia may contribute to the onset and progression of ARPC through the following mechanisms.

First, microangiopathy. Prolonged hyperglycemia may lead to thickening of the basement membrane of dermal microvessels and impaired microcirculation, which in turn can cause chronic hypoxia in skin tissue.^[9,10]^ Hypoxia not only leads to abnormal collagen metabolism and structural damage but also reduces the biomechanical tolerance of dermal tissue, making it easier for minor external forces – such as scratching – to cause basement membrane rupture and tissue damage.^[9,10]^ Second, abnormal collagen cross-linking. Chronic hyperglycemia drives oxidative stress and the massive production of advanced glycation end products (AGEs). AGEs modify the lysine and arginine residues of collagen (particularly Types I and III) via covalent bonding, thereby forming AGE-denatured collagen with receptor-activating properties.^[7,11]^ This process not only alters the physicochemical properties of collagen but also renders it a target that keratinocytes recognize abnormally, providing a key molecular substrate for the occurrence of the perforation phenomenon.^[12]^ Third, impaired wound healing. Microcirculatory disorders associated with diabetes can lead to local hypoxia and insufficient nutrient supply at the wound site, which hinders tissue repair. Abnormal collagen cross-linking caused by AGEs can make the matrix dense, rigid, and difficult to degrade, thereby forming a physical barrier, and can also inhibit cell adhesion, thus hindering the ingrowth of new granulation tissue.^[13]^ In addition, transforming growth factor-beta 3 (TGF-β3) is overexpressed in ARPC lesions, and this phenomenon is more pronounced in patients with concomitant diabetes.^[12]^ Overexpression of TGF-β3 disrupts the homeostatic balance between matrix metalloproteinases and their tissue inhibitors, thereby driving abnormal extracellular matrix metabolism, delaying epidermal regeneration, and ultimately impairing wound healing.^[12]^

The patient has a complex medical history, including T2DM, CHD, atrial fibrillation, and hypertension. After admission, investigations also revealed dyslipidemia and atherosclerosis. It is worth noting that this patient had long-standing poor glycemic control, and the appearance of typical skin lesions (erythema, papules, and nodules on the lower limbs) was temporally linked to a significant deterioration in glycemic control. Based on the literature and the characteristics of this case, we consider hyperglycemia to be one of the key triggering and aggravating factors, which may contribute to the pathogenesis through mechanisms such as microangiopathy, AGE-mediated abnormal collagen cross-linking, and impaired wound healing.

### 3.2. Clinical and pathological features

ARPC typically occurs on the extensor surfaces of the limbs and trunk and has a predilection for the lower limbs. Typically, the lesions are keratotic papules or nodules with central umbilication and a keratinous plug, accompanied by severe pruritus.^[4,14]^ Of note, Koebnerization occurs in approximately 32–56% of cases.^[4,14]^ The characteristic feature on histology is a cup-shaped epidermal depression. This structure contains a keratin plug, inflammatory cells, and degenerated collagen.^[6,7]^ The epidermis at the base of the plug is markedly thinner, and vertically oriented degenerated collagen fibers cross it. In addition, hyperkeratosis and acanthosis are seen on the margins of the cup-shaped depression, and inflammatory cell infiltration of the superficial dermis is observed.^[3,6,7]^ Special stains are useful for diagnosis: VG or Masson’s trichrome staining clearly shows degenerated collagen fibers (stained red or blue, respectively) that extend from the dermis through the defect in the epidermis.^[3]^ The skin signs can change from one stage of the disease to another, making diagnosis difficult. Thus, combining the clinical features and histopathological findings is important for diagnosis. Diagnosis is based on the criteria defined by Faver et al. (1994), which include histopathological evidence of transepidermal elimination of necrotic, basophilic collagen; clinical finding of umbilicated papules/nodules with a central, adherent keratin plug; and onset at 18 years of age or older.^[15]^ Additional supportive evidence is the presence of both the Koebner phenomenon and pruritus.^[15]^

In addition to being differentiated from common pruritic dermatoses such as prurigo nodularis and keratotic eczema, ARPC must also be distinguished histopathologically from other perforating dermatoses, primarily the following three: Kyrle’s disease: Pathologically, the condition is characterized by hyperkeratotic and parakeratotic keratin plugs penetrating through the epidermis into the dermis, accompanied by a granulomatous inflammatory reaction; denatured collagen is not the primary excreted material.^[16]^ Perforating folliculitis: Its pathological features include dilated follicular orifices filled with keratin plugs, within which curled hairs and hair shaft remnants are visible; the discharge consists primarily of follicular keratin.^[17]^ Elastosis perforans serpiginosa: The core pathological feature is the extrusion of abnormal elastic fibers – rather than collagen components – from the dermis through the epidermis.^[18]^

The patient was an elderly female with typical clinical features: skin lesions occurred mainly on the extensor surfaces of both lower limbs and presented as papules or nodules with central depressions containing a plug of keratin. Importantly, histopathological examination revealed that collagen fibers protruded through the epidermis. Therefore, the diagnosis of ARPC was clear.

### 3.3. Treatment considerations

Currently, there is no standardized treatment protocol for ARPC, and the management focuses on treating underlying systemic diseases and providing symptomatic treatment. On the one hand, it’s critical that potential systemic triggers are actively corrected. In the case at hand, hyperglycemia was the key player: strict glycemic control can reduce AGE formation and alleviate oxidative stress, which may help to delay collagen damage.

On the other hand, a multifaceted strategy for treating skin lesions is needed, including control of inflammation, relief of itching, and prevention of secondary infection. In regard to inflammation control, topical corticosteroids are an effective means of managing inflammation in the superficial layers of the dermis. Intralesional corticosteroid injections or narrowband ultraviolet B could be options for refractory or extensive lesions. In terms of pruritus, it is tightly linked to the μ-opioid receptor/IL-31 axis and Th2-related cytokines.^[19,20]^ Antihistamines are first-line treatments, although in refractory cases, biologics such as nemolizumab,^[21]^ and dupilumab^[19]^ may be considered. Additionally, Janus kinase (JAK) inhibitors (e.g., baricitinib,^[22]^ upadacitinib,^[23]^ tofacitinib^[24]^) can also help treat inflammation and pruritus. To prevent secondary infection, the skin should be cared for appropriately; the patient should be advised to avoid scratching, and topical antimicrobial agents should be used, if needed, with careful monitoring for signs of infection, especially in diabetic patients. We chose a personalized treatment approach using systemic antihistamines in addition to topical corticosteroids, avoiding biologic drugs or JAK inhibitors, considering drug availability, economic factors, and the patient’s overall condition.

The treatment outcomes in this case were satisfactory, suggesting that a dual strategy combining systemic and local approaches is effective. This treatment approach is consistent with therapeutic principles and provides a reference for the management of similar cases.

## 4. Conclusions

ARPC is a rare dermatosis, and most studies suggest that it is closely associated with chronic renal failure and diabetes.^[25,26]^ This case suggests that even in the absence of renal dysfunction, persistent hyperglycemia may contribute to the pathogenesis of ARPC through mechanisms such as AGE formation, oxidative stress, and microangiopathy. However, further research is needed to verify this. In light of this, clinicians may consider ARPC lesions as a cutaneous clue suggesting metabolic disorders. When it appears or worsens, a comprehensive assessment of the patient’s overall condition (especially glycemic control) should be conducted to detect potential systemic diseases at an early stage. Finally, treatment should involve symptomatic management of skin lesions, based on active correction of systemic contributing factors such as hyperglycemia.

## Author contributions

**Resources:** Li Li.

**Writing – original draft:** Li Li, Liuyan Zhao.

**Writing – review & editing:** Hanpeng Wu, Liuyan Zhao.
